# Flexural Creep Behavior of High-Density Polyethylene Lumber and Wood Plastic Composite Lumber Made from Thermally Modified Wood

**DOI:** 10.3390/polym12020262

**Published:** 2020-01-24

**Authors:** Murtada Abass A. Alrubaie, Roberto A. Lopez-Anido, Douglas J. Gardner

**Affiliations:** 1 Department of Civil and Environmental Engineering, Advanced Structures and Composites Center, University of Maine, Orono, ME 04469, USA; rla@maine.edu; 2 School of Forest Resources, Advanced Structures and Composites Center, University of Maine, Orono, ME 04469, USA; douglasg@maine.edu

**Keywords:** viscoelasticity, WPC, HDPE, composite, wood, creep, thermoplastic, flexure, power law, modeling

## Abstract

The use of wood plastic composite lumber as a structural member material in marine applications is challenging due to the tendency of wood plastic composites (WPCs) to creep and absorb water. A novel patent-pending WPC formulation that combines a thermally modified wood flour (as a cellulosic material) and a high strength styrenic copolymer (high impact polystyrene and styrene maleic anhydride) have been developed with advantageous viscoelastic properties (low initial creep compliance and creep rate) compared with the conventional WPCs. In this study, the creep behavior of the WPC and high-density polyethylene (HDPE) lumber in flexure was characterized and compared. Three sample groupings of WPC and HDPE lumber were subjected to three levels of creep stress; 7.5, 15, and 30% of the ultimate flexural strength (Fb) for a duration of 180 days. Because of the relatively low initial creep compliance of the WPC specimens (five times less) compared with the initial creep compliance of HDPE specimens, the creep deformation of HDPE specimens was six times higher than the creep deformation of WPC specimens at the 30% creep stress level. A Power Law model predicted that the strain (3%) to failure in the HDPE lumber would occur in 1.5 years at 30% Fb flexural stress while the predicted strain (1%) failure for the WPC lumber would occur in 150 years. The findings of this study suggest using the WPC lumber in structural application to replace the HDPE lumber in flexure attributable to the low time-dependent deformation when the applied stress value is withing the linear region of the stress-strain relationship.

## 1. Introduction

**Figure 1:**
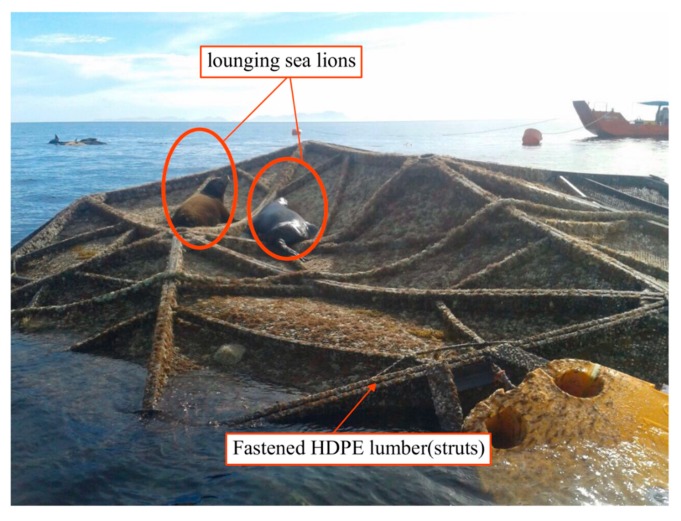
Buckled Aquapod cage made from HDPE lumber and netting (covered with biofouling) with two lounging sea lions on the exposed struts [1].

Wood plastic composites (WPCs) are commonly used as deck boards and railings thanks to their low maintenance and high durability compared with conventional pressure-treated lumber [1]. However, extensive efforts have been made to expand the use of WPCs to include structural applications [2,3,4,5,6,7,8] because of their mechanical properties, longer lifetime, and their competing commercial prices with conventional types of lumber [2,3,5,9,10]. Furthermore, WPCs made from thermally modified wood have shown potential to be used in structural applications, since they have been shown to exhibit relatively low time-dependent deformation under sustained flexural loads [11,12]. Likewise, plastic lumber is also used in low-cost structural applications. One type of plastic lumber, high-density polyethylene (HDPE) lumber, is used in the construction of aquaculture-offshore fish cages (a.k.a. Aquapod Net Pen cages) [13,14], however, the HDPE lumber experienced damage during its service life attributable to exposure to severe ocean conditions (wave action and high temperatures during the summer, ca. 48 °C in the Gulf of Mexico [14]) when these cages are partially exposed to air [14], and lounging sea lions causing damage to the exposed struts of the cage structure (in the partially exposed cages) [15,16,17], as shown in Figure 1.

The need to have a material that has a reasonable cost for the construction of aquaculture cages that also exhibits satisfactory structural performance during the service life of these cages [11,12] suggests that WPC lumber can be considered a potential alternative to HDPE lumber [11,12]. Although WPCs have been explored for use in structural applications, the material’s long-term behavior is still a subject of concern among researchers and end-users, especially in marine applications. WPC lumber exhibits viscoelastic behavior. When a constant stress is applied to a viscoelastic material, the sum of the elastic strain (instantaneous strain) and the time-dependent strain will represent the total strain (creep strain) of the viscoelastic composite material [18,19]. One dimensional (1D) viscoelastic models [power law, Maxwell, Kelvin, Prony series, and four element viscoelastic models] have been used in previous studies to describe both the short, and long-term creep-behavior of viscoelastic materials [2,3,20,21]. Alrubaie et al. [12] implemented a 1D power law viscoelastic model to describe the 180-day creep behavior of WPC lumber made from thermally modified wood with a span L = 853 mm in 4-point bending (flatwise). The power law model among other models were investigated in a preliminary study that has shown a good agreement with the short and long-term creep behavior of WPC and the HDPE lumber. Alvarez-Valencia [3] conducted a full-scale 90-day creep rupture in 4-point bending of a Z-shape WPC sheet piling with 4.70 m in length, to evaluate the time-dependent structural behavior of the WPC sheet piling, and the 1D Findlay’s power law model was used to predict the creep behavior of the WPC sheet piling that has shown good agreement with experimental data. Dura [7] conducted one, seven, and 15-day creep experiments on WPC dumbbell-shaped tensile specimens at 15, 30, and 45% of the average tensile strength, to evaluate the time-dependent behavior of the WPCs. In addition to the creep in tension, Dura [7] also conducted creep tests in compression at the same stress levels used for the tensile creep experiments, but with respect to the average maximum compression stress and to the same creep duration. Many researchers [3,6,7] have studied the large-scale flexural creep behavior of WPC specimens (i.e., when the WPC specimens have length to span ratios (L/h) that exceed the ratio recommended by the Standards [22]). Dura [7] conducted a 90-day flexural creep experiments (edgewise) on WPC specimens with a span length of 2515 mm with and without a layer of fiber reinforced polymer layer (FRP) and their creep behaviors were reported. [7]. Dura used the experimental response to verify a nonlinear 1D long-term viscoelastic model [7]. Alvarez-Valencia [3] conducted a flexural creep rupture experiment on Z-shape WPC sheet pile with a span length of 4700 mm subjected to 55% of the flexural load at failure (11.7 kN). Hamel [6] performed a three-year tensile creep test experiment on WPC dumbbell shaped specimens subjected to two different levels of stress, 20% and 50% of the average maximum stress at failure, to predict the creep behavior of 2.13 m WPC boards in flexure. Hamel [23] developed a 2D finite element (FE) model that predicted the flexural creep behavior (edgewise) based on the uniaxial quasi-static testing using the Abaqus [24] software.

The two objectives of the research presented here were: (1) to experimentally characterize the long-term (180 days) flexural creep behavior (flatwise) of WPC lumber made from thermally modified wood and compare it with the flexural creep behavior of HDPE lumber currently used in the construction of aquaculture fish cages (Aquapod Net Pen cages), and (2) to implement a power law model to describe the long-term viscoelastic creep behavior of WPC and HDPE lumber in flexure (flatwise) for a duration of 180 days, respectively. Furthermore, the model was implemented to predict the failure occurrence at the outer fiber of the WPC and HDPE lumber for a duration longer than the 180 days.

In this study, thirty 4-point bending creep frames (flat wise) located in a climate control creep room in the Advanced Structures and Composite Center (ASCC) at the University of Maine (Orono, ME, USA) were utilized to conduct 180-day creep experiments in 4-point bending (flatwise) of the WPC and HDPE lumber subjected to three different levels of stresses and each level of stress was applied to five specimens (i.e., the total number of WPC and HDPE specimens is 30).

## 2. Experimental

### 2.1. Material

**Figure 2:**
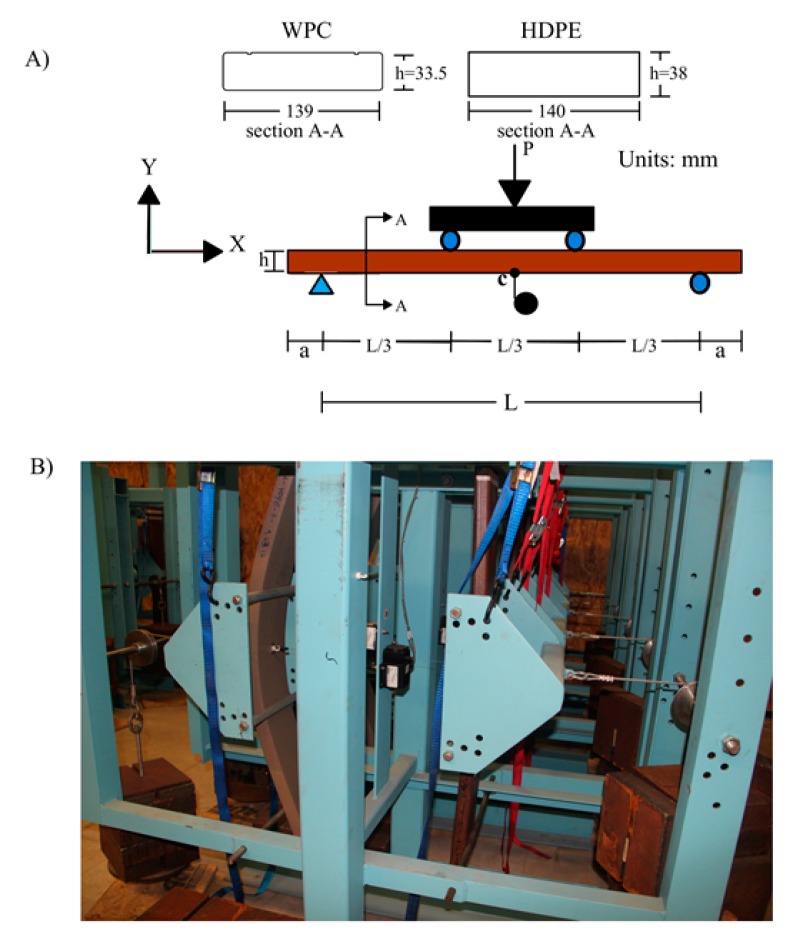
(**A**) Four-point bending test configuration used for both quasi-static tests and creep tests, (**B**) Creep frames experimental setup.

The WPC lumber with cross section dimensions [width (w), thickness (h)], (139 mm, 33.5 mm) was produced using a twin-screw Woodtruder^TM^ (Davis-Standard, Orono, Maine, USA) in the ASCC at the University of Maine (Orono, ME) [20]. The WPC lumber cross section has two grooves along the longitudinal direction (extrusion direction) of the lumber at the top layer with 3 mm width and 1.8 mm depth, and these grooves are located at 21.9 mm from the short edges of the WPC lumber, as shown in the cross-section A-A in Figure 2A. The WPC examined here is based on a patent-pending formulation, in accordance with the International Publication Number WO 2018/142314 A1 dated in 09 August, 2018, combining thermally modified wood flour (as a cellulosic material) that has been produced at Uimaharju sawmill in Finland and a high strength styrenic copolymer system (high impact polystyrene (HIPS) and styrene maleic anhydride (SMA)) in an equivalent weight ratio to each of the two constituents. Section A-A in Figure 2A shows the cross section of WPC and HDPE lumber. However, a simplifying assumption was made to consider the WPC cross-section is a rectangular cross-section and eliminate the grooved areas at the top layer in the computations. The commercially available HDPE lumber has a rectangular cross section with the width of 140 mm and the thickness of 38 mm is used in the construction of the Aquapod Net Pen cages and was provided by InnovaSea [11], to conduct this study.

### 2.2. WPC and HDPE Sample Preparation

WPC and HDPE lumber specimens with cross section dimensions (width, thickness), (139 mm, 33.5 mm) and (140 mm, 38 mm), respectively, were cut to an adequate length to fit the span of the creep test rig, L = 853 mm with an appropriate overhang at each support of the test rig [51 mm at each overhang (a) in Figure 2A], as shown in Figure 2B. To achieve the magnetic mounting of the string potentiometer that measures the creep deflection to the mid-span of the specimens, a 3-min flame treatment to each specimen followed by application of a 5-min epoxy to adhere a square metal piece (19 × 19 mm) to the mid-span of each specimen (flatwise). Thereafter, a magnetic hook was mounted on the square metal and the string potentiometer was attached to the hook during the creep loading, and hence, the creep mid-span deflection was acquired, accordingly.

### 2.3. 180-Day Creep Experimental Setup

Prior to the creep loading and in accordance with ASTM D618 [23], WPC and HDPE specimens were preconditioned in the climate control creep room at the ASCC for one week. Thereafter, and according to ASTM D6109 and ASTM D6815 [25], the long-term WPC and HDPE specimens were loaded in 4-point bending (flat wise) with values of L/h 22 and 20, respectively. The relative humidity (RH) and temperature were controlled during the 180 days of the creep experiment to be 50 ± 5% and 21 ± 2 °C. The crosshead speed used to load the WPC and HDPE specimens for creep was the same crosshead speed used in the quasi-static testing to obtain the mean ultimate flexural stress (i.e., to ensure the initial applied loading will be applied to the specimens not less than one minute and not greater than 10 min). The measurements and the recordings of the; applied flexural level, creep displacements, and the relative humidity and the temperature of the climate control creep room, are managed by a data acquisition system (DAQ) located at the climate control room at the ASCC at the University of Maine. 

Based on the applied flexural stress level relative to the flexural strength (Fb), the WPC and HDPE specimens have been divided into three groups: 7.5% of Fb, 15% of Fb, and 30% of Fb, respectively. The selection of the stress levels was made based on the level of the linear region which is below 40% of the ultimate flexural strength in the stress strain relationship in flexural tests specified in ASTM D6109, to avoid the failure occurrence during the creep duration if the selected levels of stress were higher than 40% of the ultimate flexural strength.

### 2.4. Quasi-Static Tests

To obtain the apparent elastic modulus (E) and the mean of the flexural strength, five specimens of each of the WPC and HDPE lumber were cut with a span to depth ratio 16:1 with an adequate overhang length over the supports of the fixture, and were tested in 4-point bending in accordance with ASTM D6109 [21], as shown in Figure 2A. The support spans of the WPC and HDPE specimens were L = 545 mm and L = 620 mm, respectively. The crosshead rate used on the WPC and the HDPE specimens during the 4-point bending test were selected in accordance with ASTM D 6109 [22], to be 15.9 and 18 mm/min, respectively. For the 180-day creep experiments, three levels of flexural creep stress were applied to the WPC and HDPE specimens (five specimens in each level). These three levels were: 7.5%, 15%, and 30% of the mean of the flexural strength obtained from the quasi-static tests. The flexural test was conducted in accordance with ASTM D6109. The flexural stress versus strain relationships of the WPC and HDPE lumber used in this study were reported elsewhere [26,27] The selection of the stress levels was made based on; (1) the use of the WPC and HDPE lumber in submerged Aquapod Net Pen cages is expected to be under low stresses (the structural members of the cage does not carry the weight of the cage, except to withstand the mooring and the buoyancy system [14,15,28], (2) researchers in previous studies [6,7,20,29,30,31] have studied the creep behavior of WPCs under stress levels that were greater than or equal to 30% and recommended further studies using low stress levels [6,32], thus, it is important to investigate the creep behavior of WPCs under low stress levels. Table 1 shows the values of the apparent elastic modulus of the WPC specimens and the HDPE with their standard deviation values and the selected levels of the creep flexural stress. The determination of the apparent elastic modulus of WPC and HDPE specimens was performed in accordance with ASTM D6109 [22], by computing the slope of the line obtained from the linear regression to the linear portion in the load-midspan deflection curve. Since the span to depth ratio (L/h) of the tested WPC and HDPE specimens was 16 which met the recommended L/h in the ASTM standards, the shear deformation was ignored in the computation of the apparent elastic modulus (further discussion on shear deformation in the computation of the elastic modulus of the WPCs with similar formulation was described elsewhere [11,12]). Then, the flexural strength (Fb) was determined: (1) for WPC, as the ultimate flexural stress at midspan at failure, (2) for HDPE, as the flexural stress at midspan corresponding to 3% of outer fiber strain. The results are reported in Table 1. The mechanical properties of the HDPE lumber tested in this study agreed with the mechanical properties reported in the data sheet of the manufacturer [33]. In accordance with ASTM D6109, the flexural strength is determined as the maximum stress in the outer fibers at failure or when the strain in the outer fibers equals 3%, whichever occurs first.

## 3. Results and Discussion

### 3.1. Determination of the Creep Stress Levels

The applied flexural stress levels for WPC and HDPE lumber were selected to be as percentages of the mean of the flexural strength obtained from the quasi-static tests, Fb = 41.2 MPa, and Fb = 14.1 MPa, respectively. Thus, the flexural creep stress levels applied on the three groups of each of WPC and HDPE lumber were approximately 7.5%, 15%, and 30% of the ultimate flexural strength, as shown in Table 1. Since the cross section of the WPC lumber has a depth (d) which is 88% of the depth of the HDPE lumber and according to ASTM D 6109 the expected mid-span creep displacement of the WPC lumber is expected to be 14% higher than the mid-span creep displacement of the HDPE lumber under the same applied stress with the assumption that the both materials have the same strength and elastic modulus. Thus, to ignore this difference in the cross section of each materials, the applied creep stresses were selected to be approximately at the same level to each group of WPC and HDPE lumber, as percentages of the flexural strength of each material (Table 1). The applied stresses to each group of HDPE lumber is approximately 14% higher than the applied stresses of each group of WPC lumber. This difference was applied to overcome the difference between the cross section (depth) of the WPC lumber and the cross section (depth) of the HDPE lumber. However, each group of HDPE and WPC lumber was given a name based on the applied stress to be; group 7.5%, group 15%, and group 30%.

### 3.2. Experimental Comparison Between the Long-Term Creep of WPC and HDPE Lumber

**Figure 3:**
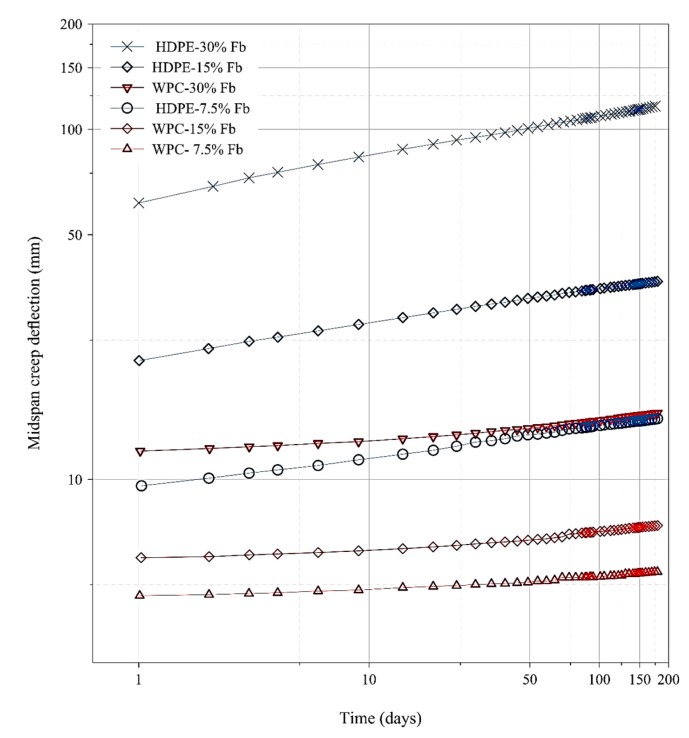
Time-dependent mid-span creep displacement for WPC and HDPE specimens at different stress levels as percentages from the flexural strength Fb.

Three levels of stress were applied on each group of five specimens of WPC and HDPE lumber. The mean of the mid-span creep deflection of each group of WPC and HDPE lumber was reported, as shown in the log-log space axes in Figure 3.

In accordance with ASTM D 6815 [25], the acceptance criteria of the creep behavior of the specimen is evaluated via: (1) the decrement in the creep rate (all the subsequent creep rate data should be decreasing during the duration of the creep test), (2) the fractional deflection (FD) should not exceed 2, which is obtained from dividing the mid-span creep deflection at the end of the creep experiment by the initial mid-span deflection (D0) [25]. The values of initial midspan displacement measured during the first four minutes of the creep test and were reported in Table 2. In addition to D0, the initial strain (ε0) was reported in Table 2. The computation of the initial strain was made in accordance with ASTM D 6109. The creep rate in this study was measured at each 30 days as reported in Table 3. Table 3 shows the 30-day creep rate of the three groups of each of WPC and HDPE specimens during the 180-day creep experiment. It can be seen that the values of the WPC fractional deflection under the three different flexural stress levels were within the acceptable limit recommended by ASTM D 6815, whereas, the values of the HDPE fractional deflection failed to meet the recommended fractional deflection limit. However, all the WPC and HDPE groups exhibited a decreasing creep rate during the 180-day creep experiment as reported in Table 3, except a noticeable increase in the creep rate of the HDPE group-15% Fb for the time between the 150 and 180 days. This increase can be attributable to the assumption that the creep of HDPE specimens entered the steady-state of creep in the secondary region [34].

**Figure 4:**
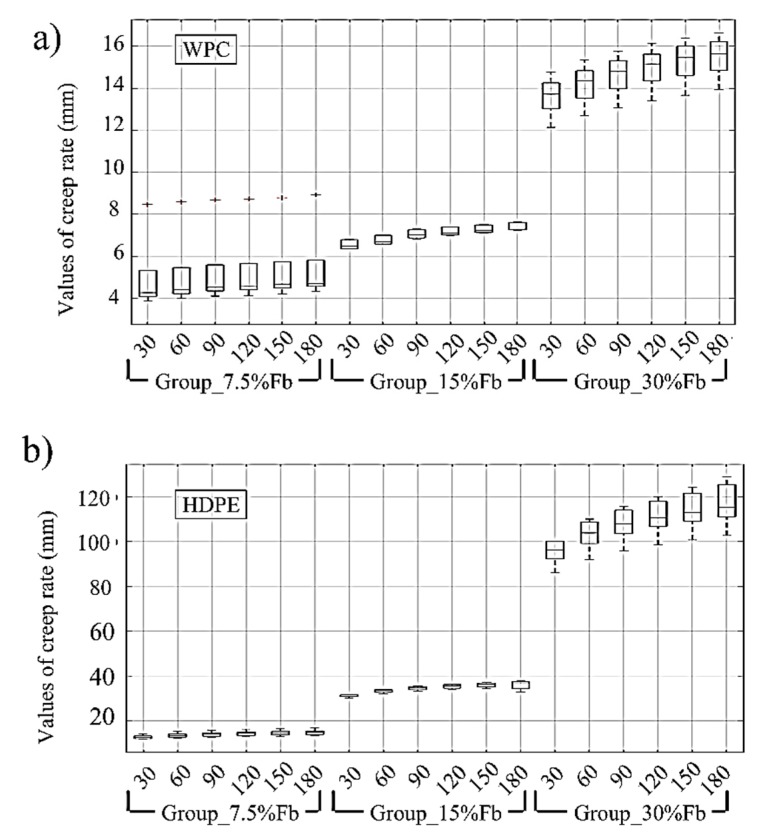
(**a**) Statistical analysis of variance (ANOVA) that investigates the reduction in creep rate of the WPC specimens subjected to three applied flexural creep stress levels. (**b**) ANOVA that investigates the reduction in creep rate of the HDPE specimens subjected to three applied flexural creep stress levels as percentages from the flexural strength Fb.

For further comparison between the creep behavior of WPC and HDPE specimens, a statistical analysis of variance (ANOVA) study of the mid-span creep deflection of each specimen at each group of the WPC and HDPE was conducted and the results are shown in Figure 4. At the applied flexural stress level of 7.5% of the flexural strength, HDPE specimens showed a mid-span creep deflection exceeding two times the mid-span creep deflection of the WPC specimens. As the levels of applied flexural stress increased from 7.5% to 15% and 30%, the HDPE specimens showed mid-span creep deflections exceeding five times and seven times the mid-span creep deflection of the WPC at the same applied flexural levels of stress, respectively. The rate of increase in the mid-span creep deflection between the HDPE specimens subjected to 7.5 and 15% (i.e., HDPE specimens for-7.5% Fb, and 15% Fb) of the flexural strength was below 150%, whereas it was below 35% for the WPC specimens (WPC specimens in group-7.5% and 15% of Fb). When the applied flexural stress levels increased from 15% to 30% of the flexural strength, the creep rate between groups-15% and 30% of Fb was below 215% for the HDPE specimens, and below 110% for WPC specimens. This low time-dependent mid-span deflection creep behavior of the WPC specimens compared with the behavior of HDPE specimens can be anticipated based on their initial compliances (the reciprocal of the elastic modulus); 0.232 GPa-1 and 1.11 GPa-1, respectively. In regards to the comparison of the time-dependent viscoelastic behavior of the WPC with the WPC in previous studies; a short-term time-dependent behavior comparison of the WPC with the same formulation of WPC in this study was presented elsewhere [11], and Alrubaie et al. [12] have presented a comparison between the creep behavior of the group-30% of Fb of WPC presented in this study and the creep behavior of WPC from previous studies. Thus, a comparison to the creep behavior of the WPC used in this study with WPC material from previous studies is not discussed here.

### 3.3. Time-Dependent Creep Modeling

**Figure 5:**
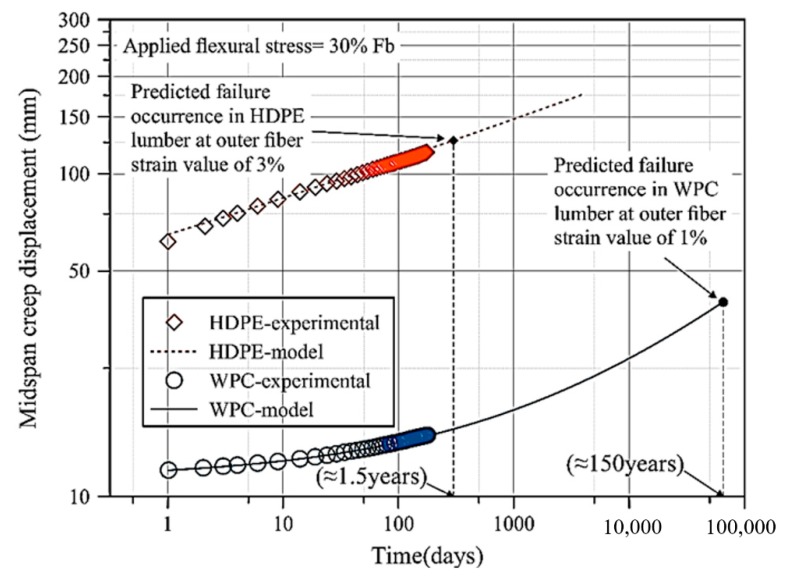
Predicted failure occurrence in the outer fiber strain of WPC and HDPE lumber for the specimens subjected to 30% Fb flexural stress using the power law model.

An empirical power law model was used to describe the 180-day mid-span flexural creep displacement. The model showed a good degree of agreement with the experimental data of the WPC and HDPE lumber in 4-point bending creep test (flatwise). Based on the assumption that the WPC should fail at a flexural strain in outer fiber of 1%, and the HDPE lumber should fail at a flexural strain in outer fiber of 3% (similar to the failure strain value mentioned in ASTM D 6109), the computed mid-span creep the predicted failure occurrence for WPC and HDPE in flexure and under a flexural stress of 30% of Fb will occur after 150 years and 1.5 years, respectively, as shown in Figure 5. To investigate the stress-independency behavior (viscoelastic behavior) of the WPC and HDPE lumber with regards the three applied stress levels (7.5%, 15%, and 30% of Fb), a power law model was implemented to describe the normalized mid-span creep displacement behavior (*d*(*t*)). Equation (1) describes the normalized midspan creep displacement behavior:

(1) $$d\left(t\right)=\frac{D\left(t\right)}{D_{0}}$$

where *d*(*t*) is the time dependent midspan deflection. For a 4-point bending test configuration, the initial mid-span creep displacement (*D*_0_) is related to the applied flexural stress, as shown in Equation (2):

(2) $$D_{0}=\frac{23}{108}\frac{F_{b}}{E}\frac{L^{2}}{h}$$

where F_b_ and *E* are the flexural stress and elastic modulus, respectively, *L* is the support span, and *h* is the depth of the WPC and HDPE specimen. The normalized mid-span creep displacement is predicted, as shown in Equation (3):

(3) $$d\left(t\right)=1+d_{1}t^{m}$$

where *d*_1_ and m are the stress-independent power law parameters. These parameters (*d*_1_ and *m*) were computed from the experimental least square error data fitting using a Matlab code. The creep behavior of HDPE lumber and WPC lumber has been predicted for ten years using the power law model, as was reported in Table 4. According to InnovaSea Systems Inc. (Morril, Maine, USA), the estimated service life of aquaculture cages is ten years. The prediction showed the failure occurrence (maximum strain at outer fiber layer) will not occur for both WPC and HDPE specimens for the stress levels 7.5% and 15% of Fb. Whereas, the failure occurrence was predicted in 1.5 years for the HDPE lumbers subjected to 30% of Fb. For this reason, WPCs are considered in the construction of aquaculture cage structures subjected to stress levels 30% below Fb.

**Figure 6:**
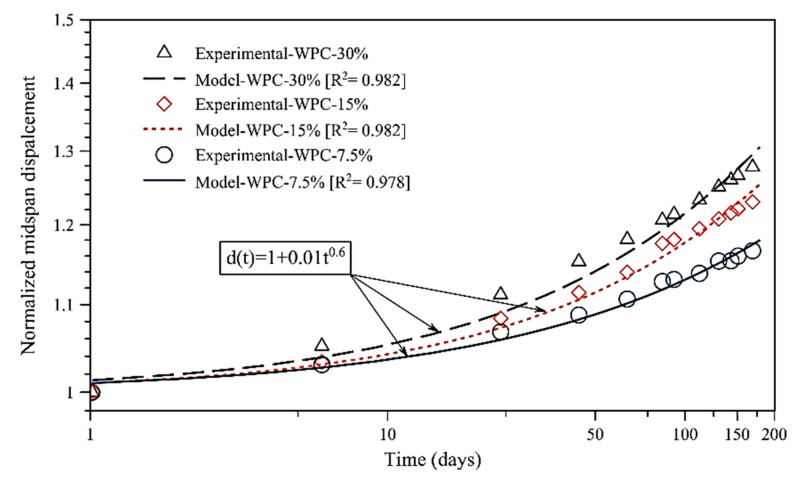
Comparison of power law model and experimental creep result for WPC lumber.

**Figure 7:**
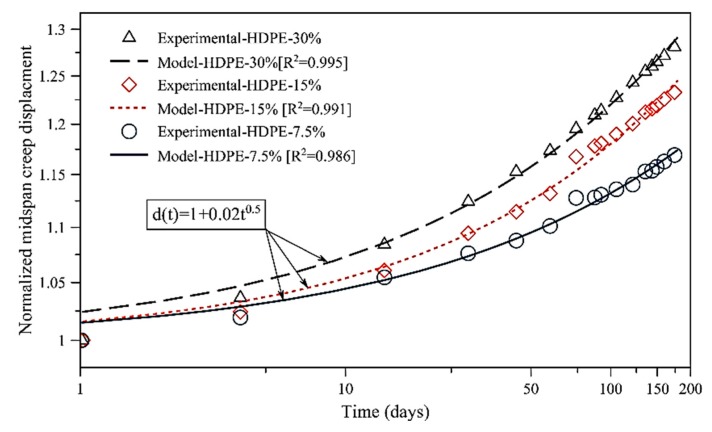
Comparison of power lay model and experimental creep results for HDPE lumber.

Values of the normalized mid-span creep displacement are reported in Table 5. The normalized power law model showed the stress-independency [18] of the WPC and HDPE lumber by having similar values of the normalized power law model (*d*_1_ and *m*) at different flexural stress levels, respectively. Figure 6 and Figure 7 illustrate the stress-independency behavior of each group of the WPC and HDPE lumber via describing the normalized mid-span creep displacement by the normalized creep behavior.

## 4. Conclusions

The WPC in this study showed a reduced time-dependent creep behavior compared to HDPE. WPCs thus show potential to replace HDPE lumber in the construction of aquaculture cage structures. While previous studies have studied the creep behavior of WPC at relatively high stress levels, this study conducted the creep experiments using levels of stresses that were below 30% of the ultimate flexural strength, which are typical for the intended design application. During the comparison between the creep behavior of WPC and HDPE specimens at the low stress levels (7.5% and 15% of Fb), the fractional deflections (FD) of HDPE were 122% and 192% higher than the FD of the WPC specimens, respectively. Whereas, the FD of HDPE specimens at 30% stress level was 300% higher than the FD of the WPC specimens. This can be advantageous for using WPC lumber as a replacement of the HDPE lumber in the construction of aquaculture cages.

The power law model was a useful tool to describe and predict the creep behavior of both WPC and HDPE lumber for all the stress levels (7.5%, 15%, and 30% of Fb). This model predicted that both HDPE lumber and WPC lumber show low creep rate during ten years at stress levels below 15% of Fb. Whereas, at stress level 30% of Fb, failure occurrence at outer fiber is predicted to happen at 1.5 years for HDPE lumber and at 150 years for WPC lumber.

## Figures and Tables

**Table 1:** Values of elastic modulus (E), flexural strength, and the applied creep stress level of WPC and HDPE lumber obtained from 4-point quasi-static testing.

Material	Name of the Group	Applied Stress Level	E (GPa)	Mean Fb (MPa)	Applied Flexural Creep Stress Level (MPa)
WPC	group 7.5% Fb	7% Fb	4.34 ± 0.26	41.2 ± 4.53	3.0 ± 0.08
	group 15% Fb	14% Fb			5.9 ± 0.04
	group 30% Fb	29% Fb			11.8 ± 0.09
HDPE	group 7.5% Fb	8% Fb	0.93 ± 0.03	14.1 ± 0.70	1.1 ± 0.05
	group 15% Fb	16% Fb			2.2 ± 0.04
	group 30% Fb	31% Fb			4.4 ± 0.09

**Table 2:** Initial midspan deflection (D_0_) and strain (**ε_0_**) of WPC and HDPE lumber at three different stress levels.

Material % of Fb	D_0_ (mm)	ε_0_ (%)
WPC-7.5%	2.96	0.1
WPC-15%	5.8	0.2
WPC-30%	11.3	0.3
HDPE-7.5%	5.5	0.2
HDPE-15%	8.74	0.3
HDPE-30%	18.71	0.6

**Table 3:** Values of creep rate deflection (D) (mm) of all the groups of WPC and HDPE specimens at 30th, 60th, 90th, 120th, 150 and 180th day respectively and the fractional deflection (FD) at the 180th day with respect to the initial deflection D0.

Creep Rate and FD	Material-% of Fb					
	WPC-7.5%	WPC-15%	WPC-30%	HDPE-7.5%	HDPE-15%	HDPE-30%
D_30_-D_0_	0.54	0.99	2.35	7.31	15.57	72.54
D_60_-D_30_	0.13	0.21	0.57	0.77	1.72	7.80
D_90_-D_60_	0.12	0.28	0.45	0.51	1.04	4.62
D_120_-D_90_	0.05	0.12	0.35	0.36	0.65	3.45
D_150_-D_120_	0.08	0.12	0.29	0.25	0.5	2.87
D_180_-D_150_	0.06	0.09	0.23	0.22	0.62	2.47
**FD_180_**	1.22	1.33	1.28	2.71	3.88	5.11

**Table 4:** 10-year prediction of the creep displacement of the WPC and HDPE lumber (in accordance with ASTM D6109).

Material Name-% of Fb	Outer Fiber Strain at Failure %	Mid-Span Displacement at Failure (mm)	Predicted Mid-Span Creep Displacement in 10 Years (mm)
WPC-7.5%	1.040	46	6
WPC-15%			11
WPC-30%			22
HDPE-7.5%	3.004	120	21
HDPE-15%			50
HDPE-30%			165

**Table 5:** Power law model parameters.

Material Type	Model Parameters	
	*d*_1_	*m*
WPC	0.011	0.596
HDPE	0.018	0.494

## References

[1] Klyosov A.A. (2007). Wood-Plastic Composites.

[2] Slaughter A.E. (2004). Design and fatigue of a structural wood-plastic composite. Master of Science thesis.

[3] Daniel A.-V. (2010). Structural Performance of Wood Plastic Composite Sheet Piling. J. Mater. Civil Eng..

[4] Gardner D., Han Y. Towards Structural Wood-Plastic Composites: Technical Innovations. Proceedings of the 6th Meeting of the Nordic-Baltic Network in Wood Material Science and Engineering (WSE).

[5] Haiar K.J. (2000). Performance and Design of Prototype Wood-Plastic Composite Sections.

[6] Hamel S.E. (2011). Modeling the Time-Dependent Flexural Response of Wood-Plastic Composite Materials. Ph.D. Thesis.

[7] Dura M.J. (2005). Behavior of Hybrid Wood Plastic Composite-Fiber Reinforced Polymer Structural Members for Use in Sustained Loading Applications. Master’s Thesis.

[8] Melissa K. (2006). Structural Design of Hollow Extruded WPC Sheet Piling. Master’s Thesis.

[9] Bright K.D., Smith P.M. (2007). Perceptions of New and Established Waterfront Materials by US Marine Decision Makers. Wood Fiber Sci..

[10] Tamrakar S., Lopez-Anido R.A. (2011). Water Absorption of Wood Polypropylene Composite Sheet Piles and Its Influence on Mechanical Properties. Constr. Build. Mater..

[11] Alrubaie M.A., Lopez-Anido R.A., Gardner D.J., Tajvidi M., Han Y. (2019). Experimental investigation of the hygrothermal creep strain of wood–plastic composite lumber made from thermally modified wood. J. Thermoplast. Compos. Mater..

[12] Alrubaie M.A., Lopez-Anido R.A., Gardner D.J., Tajvidi M., Han Y. (2019). Modeling the hygrothermal creep behavior of wood plastic composite (WPC) lumber made from thermally modified wood. J. Thermoplast. Compos. Mater..

[13] InnovaSea Systems, Inc. A4700 BRIDLE SYSTEM IN GRID MOORING CELL. 2016 [cited September 2017]. www.innovasea.com.

[14] Vandenbroucke K., Metzlaff M. (2013). Abiotic stress tolerant crops: Genes, pathways and bottlenecks. Sustainable Food Production.

[15] InnovaSea Systems, Inc. (2015). Report on Structural Damage to A4800 AquaPod.

[16] Gardner D.J. (2015). Development of Structural Wood Plastic Composite Timber for Innovative Marine Application. Research Reinvestment Funds (RRF) Seed Grant Program.

[17] Commerce D.O. Water Temperature Table of All Coastal Regions. https://www.nodc.noaa.gov/dsdt/cwtg/all.html.

[18] Gibson R.F. (2016). Principles of Composite Material Mechanics.

[19] Barbero E.J. (2013). Finite Element Analysis of Composite Materials Using AbaqusTM.

[20] Tamrakar Sandeep R.A.L.-A., Kiziltas A., Gardner D.J. (2011). Time and temperature dependent response of a wood–polypropylene composite. Compos. Part A.

[21] Pooler D.J. (2001). The Temperature Dependent Non-Linear Response of a Wood Plastic Composite.

[22] ASTM International (2013). Standard Test Methods for Flexural Properties of Unreinforced and Reinforced Plastic Lumber and Related Products, D6109-13.

[23] Hamel S.E., Hermanson J.C., Cramer S.M. (2014). Predicting the flexure response of wood-plastic composites from uni-axial and shear data using a finite-element model. J. Mater. Civ. Eng..

[24] Abaqus/CAE (2017). Computer Software.

[25] ASTM International (2015). Standard Specification for Evaluation of Duration of Load and Creep Effects of Wood and Wood-Based Products, D6815-09 (Reapproved 2015).

[26] Alrubaie M.A. (2019). Investigating the Time-dependent and the Mechanical Behavior of Wood Plastic Composite Lumber Made from Thermally Modified Wood in the Use of Marine Aquacultural Structures. Ph.D. Thesis.

[27] Alrubaie M.A.A., Gardner D.J., Lopez-Anido R.A. (2020). Structural Performance of HDPE and WPC Lumber Components Used in Aquacultural Geodesic Spherical Cages. Polymers.

[28] Page S.H. (2013). Aquapod Systems aquaculture Aquapod systems for Sustainable Ocean Aquaculture Aquaculture. Sustainable Food Production.

[29] King D., Hamel S. The Tensile Creep Response of a Wood-Plastic Composite in Cold Regions. Proceedings of the 10th International Symposium on Cold Regions Development.

[30] Chassagne P., Saïd E.B., Jullien J.F., Galimard P. (2005). Three dimensional creep model for wood under variable humidity-numerical analyses at different material scales. Mech. Time-Depend. Mater..

[31] Hamel S.E., Hermanson J.C., Cramer S.M. (2013). Mechanical and time-dependent behavior of wood–plastic composites subjected to tension and compression. J. Thermoplast. Compos. Mater..

[32] Chang F.-C. (2011). Creep Behaviour of Wood-Plastic Composites.

[33] Tangent Technologies L. Polyforce Sturctural Recycled Plastic Lumber. 2015 [cited November 2018]. Mechanical propertied of the HDPE Polyforce Lumber. http://tangentusa.com/wp-content/uploads/2016/01/PolyForce_DataSheet_01_20_16.pdf.

[34] ASTM International (2013). Standard Test Methods for Compressive and Flexural Creep and Creep-Rupture of Plastic Lumber and Shapes, D6112-13.

